# Optimization of Probabilistic Shaping for Nonlinear Fiber Channels with Non-Gaussian Noise

**DOI:** 10.3390/e22080872

**Published:** 2020-08-08

**Authors:** Henrik Enggaard Hansen, Metodi P. Yankov, Leif Katsuo Oxenløwe, Søren Forchhammer

**Affiliations:** DTU Fotonik, Technical University of Denmark, Bygning 343, Ørsted Plads, 2800 Kongens Lyngby, Denmark; meya@fotonik.dtu.dk (M.P.Y); lkox@fotonik.dtu.dk (L.K.O.)

**Keywords:** constellation shaping, nonlinearities, fiber optic communication, coherent communications

## Abstract

Probabilistic constellation shaping is investigated in the context of nonlinear fiber optic communication channels. Based on a general framework, different link types are considered—1. dispersion-managed channels, 2. unrepeatered transmission channels and 3. ideal distributed Raman amplified channels. These channels exhibit nonlinear effects to a degree that conventional probabilistic constellation shaping strategies for the additive white Gaussian (AWGN) noise channel are suboptimal. A channel-agnostic optimization strategy is used to optimize the constellation probability mass functions (PMFs) for the channels in use. Optimized PMFs are obtained, which balance the effects of additive amplified spontaneous emission noise and nonlinear interference. The obtained PMFs cannot be modeled by the conventional Maxwell-Boltzmann PMFs and outperform optimal choices of these in all the investigated channels. Suboptimal choices of constellation shapes are associated with increased nonlinear effects in the form of non-Gaussian noise. For dispersion-managed channels, a reach gain in 2 spans is seen and across the three channel types, gains of >0.1 bits/symbol over unshaped quadrature-amplitude modulation (QAM) are seen using channel-optimized probablistic shaping.

## 1. Introduction

Future demands for data transmission applications push optical fiber communication to operate with increasingly higher spectral efficiency. Higher-order modulation is well-suited for this since it is independent of the operating bandwidth [1], but conventional quadrature-amplitude modulation (QAM) formats leave a gap to the channel capacity. Constellation shaping has the potential to close this gap through the selection of modulation symbols tailored to the characteristics of the channel in use. Both geoemtric and probabilistic schemes are possible [1]. For the classical additive white Gaussian noise (AWGN) channel, the capacity and the potential in constellation shaping are well-understood, with known optimal results [2]. In particular, the Maxwell-Boltzmann distribution is known to be a near-optimal choice of a discrete probability mass function (PMF) for constellation shaping [3] and this PMF has been successfully applied to standard optical fiber channels, such as long-haul transmission with erbium-doped fiber amplifiers (EDFA) [4].

The widely applied enhanced Gaussian noise (EGN) model for the fiber assumes that the combined effects of amplified spontaneous emission (ASE) noise and nonlinear interactions follow Gaussian statistics, with a variance dependent on the higher-order signal statistics [5,6]. It is thus tempting to use shaping schemes based upon AWGN channel-like assumptions for optical fiber channels. However, such models may lead to suboptimal shaping design beyond the standard, highly dispersive single mode fiber scenarios. This questions the assumptions of the memoryless AWGN channel, which could misleade system design for channels where the nonlinear effects are particularly prevalent [7].

In this paper, we investigate by simulations circumstances where the AWGN channel assumptions lead to a suboptimal constellation design compared to what can be achieved through optimization of the constellation PMF without assuming the distribution should be a Maxwell-Boltzmann distribution. Three scenarios of nonlinear fiber optic channels will be considered, where the channel configuration results in a relatively high influence of the nonlinear effects in the optical fiber compared to ASE noise [4,8]. The first channel is the dispersion-managed transmission system, which uses periodic dispersion compensation. Dispersion-managed systems were introduced to counter the limitations of direct-detection systems, which did not compensate for dispersion digitally. The periodic dispersion compensation recorrelates co-propagating signals and in turn enhances nonlinear distortion [1]. To the best of our knowledge, there is no work on targeting probabilistic shaping for dispersion-managed transmission.

The second channel is an unrepeatered transmission system, which features a single span of fiber with no amplification of the signal—apart from at the transmitter and receiver. At distances above 100 km, such systems require high launched signal power, which directly leads to increased nonlinear distortions [9]. Constellation shaping for unrepeatered channels was considered in Reference [10], where gains were shown using designs based upon the EGN model.

Finally, the channels with periodic and distributed Raman amplifiers are considered. Raman amplification allows for continuous signal amplification and has improved noise figures compared to conventional “lumped” amplification solutions ([11] Ch. 3). However, the continuous amplification also results in elevated signal powers for extended distances, which compounds nonlinear phase shift and nonlinear distortion. In Reference [1], ring constellations for ideal distributed Raman amplification (IDRA) was analysed in the context of capacity limits. These constellations are only of theoretical interest as they are partially continuous.

In this paper, probabilistic constellation shapes are obtained through an optimization strategy, which does not presume a certain family of probability distributions, and through a channel tailored Maxwell-Boltzmann distribution. Across the channel types, channel-tailored probabilistic shaping achieves gains of >0.1 bits/symbol over the unshaped QAM case.

In Section 2, we describe information measures, fiber propagation and relevant background for constellation shaping. In Section 3, the optimization strategy and simulation scheme used in this paper are presented. In Section 4 and Section 4.1, Section 4.2 and Section 4.3, the dispersion-managed, unrepeatered and IDRA channels are discussed and their results presented, respectively. Finally, in Section 5 and Section 6, the results are discussed and the paper is concluded.

## 2. Information Measures and Probabilistic Constellation Shaping

### 2.1. Information Measures

We will consider communication using complex-valued QAM symbols belonging to a discrete alphabet, X. A sequence is transmitted, consisting of *K* symbols, $x_{1}^{K}$, each drawn from a discrete random variable *X*, with associated PMF $P_{X}$. The communication system under consideration sends data from transmitter to receiver through a noisy channel, from which we obtain the received sequence $y_{1}^{K}$, which is a realization of the received random variable *Y*. The effective channel, including any signal processing, is thus represented by the probability density function (PDF) $p_{Y|X}$.

Mutual information (MI) is used to asses the performance of constellation shapes and it is defined by

(1) $$I\left(X;Y\right)=\lim_{K\to \infty }\frac{1}{K}I\left(X_{1}^{K};Y_{1}^{K}\right)=\lim_{K\to \infty }\frac{1}{K}\left(H\left(X_{1}^{K}\right)-H\left(X_{1}^{K}|Y_{1}^{K}\right)\right),$$

where $H(X_{1}^{K})$ and $H(X_{1}^{K}|Y_{1}^{K})$ are the information entropy and conditional entropy, respectively. The entropy term is calculated as

(2) $$H\left(X_{1}^{K}\right)=-\sum_{k=1}^{K}\log_{2}P_{X}\left(x_{k}\right),$$

thus assuming independence of the symbols and that the distributions are stationary over the whole sequence.

In order to estimate the conditional entropy, H(X|Y), the conditional probability, $P_{X|Y}$, must be known, but analytic forms are not known for the general fiber optic channel. Instead, an auxiliary channel model, $q_{y_{1}^{K}|x_{1}^{K}}$, is applied, which approximates the true channel, $p_{Y|X}$. Through this, we obtain the conditional PMF,

(3) $$Q_{x_{1}^{K}|y_{1}^{K}}=\frac{P_{x_{1}^{K}}\cdot q_{y_{1}^{K}|x_{1}^{K}}}{\sum_{x_{1}^{K}}P_{x_{1}^{K}}\cdot q_{y_{1}^{K}|x_{1}^{K}}}.$$

Using an auxiliary channel to estimate the conditional entropy, H(X|Y), results in an upper bound and thus gives a lower bound on mutual information [12]. This lower bound is called the achievable information rate (AIR), which will be the information measure used to asses the performance of the constellation shaping schemes. The tightness of the bound depends on the accuracy of the auxiliary channel compared to the actual channel [12].

### 2.2. Fiber Propagation

To simulate fiber propagation, the split-step Fourier method (SSFM) is used to numerically solve the coupled-mode nonlinear Schrödinger equation ([13], Ch. 6); given as

(4) $$\begin{array}{cc} \frac{\partial }{\partial z}E_{x}+\frac{\alpha }{2}E_{x} & =-i\frac{\beta_{2}}{2}\frac{\partial^{2}}{\partial t^{2}}E_{x}+i\gamma \left(|E_{x}{|}^{2}+\frac{2}{3}{\left|E_{y}\right|}^{2}\right)E_{x},\\ \frac{\partial }{\partial z}E_{y}+\frac{\alpha }{2}E_{y} & =-i\frac{\beta_{2}}{2}\frac{\partial^{2}}{\partial t^{2}}E_{y}+i\gamma \left(|E_{y}{|}^{2}+\frac{2}{3}{\left|E_{x}\right|}^{2}\right)E_{y}, \end{array}$$

where $E_{x}$, $E_{y}$ are the fields in two orthogonal polarizations, *z* is the position in the fiber, *t* the time and $\beta_{2}$ is group-velocity dispersion, α is the loss and γ is the nonlinear coefficient. Equation (4) only describes propagation in a fiber with dispersion and nonlinearities. Noise may be added as a Langevin-noise source term creating a stochastic nonlinear Schrödinger equation, or added to stepwise solutions to the signal field using SSFM. In the case of distributed Raman amplifiers, the noise contribution is distributed along the fiber span, where the distributed Raman generated ASE noise is included in the SSFM as in Reference [8].

Co-propagating data signals and added noise interact through chromatic dispersion and the nonlinear Kerr effect on the right hand of Equation (4), resulting in a distorted signal with stochastic influences. Neighboring wavelength-division multiplexing (WDM) channels also contribute to the stochastic effects in single WDM channel receivers or optical networks. The contribution from co-propagating signals is determinstic, but to compensate it would require the entire co-propagating WDM signal to be received and processed in unison. This would be impractical for high baudrates and impossible for optical networks using optical routing.

Albeit there is no general closed-form solution to the stochastic nonlinear Schrödinger equation, approximate models do exist, which give some insight into the interplay between signal characteristics and performance. In relation to constellation shaping, the EGN model [5] is particularly relevant, as it gives an approximate closed-form relationship between the statistical properties of the modulation symbols and nonlinear interference (NLI) noise variance, $\sigma_{\mathrm{NLI}}^{2}$ [6,14]. For the purposes of constellation shaping, the important property of $\sigma_{\mathrm{NLI}}^{2}$ is that it is proportional to higher-order moments $E[|X{|}^{4}]$ and $E[|X{|}^{6}]$, where E[·] is the expectation operator. Thus, nonlinear noise variance can be minimized by minimizing the two higher-order moments, which is achieved using phase-shift keyed modulation [4]. Minimizing $\sigma_{\mathrm{NLI}}^{2}$ will not necessarily result in maximizing MI, as ASE noise is also present in the channel.

### 2.3. Constellation Shaping

Constellation shaping alters the probability of occurrence or geometric placement of constellation symbols to match the channel characteristics thus increasing the MI. We focus on the former, referred to as probabilistic shaping. In AWGN channels, an increased Euclidean distance between symbols will increase MI. This can be achieved by lowering the probability of occurrence of high-amplitude symbols, which under a fixed power constraint, leads to an increase in Euclidean distance between symbols [3]. Through optimization, known optimum PMFs are found [15]. For discrete distributions, a frequently used PMF for probabilistic constellation shaping is the Maxwell-Boltzmann distribution, which minimizes the average energy for a given entropy [3]. Its PMF is given by

(5) $$P_{\mathrm{MB}}\left(x;\lambda \right)={\exp(-\lambda |ax|}^{2})/Z\left(\lambda \right),\;Z\left(\lambda \right)=\sum_{x\in X}{\exp(-\lambda |ax|}^{2}),$$

where λ controls the trade-off between average energy and entropy and *a* is a scaling factor set to ensure a fixed average power $P_{\mathrm{av}}=\sum_{x\in X}P_{\mathrm{MB}}\left(x;\lambda \right){\left|ax\right|}^{2}$. Note that the uniform PMF is a special case of Maxwell-Boltzmann with λ=0.

For Gaussian noise and a given signal-to-noise ratio (SNR), an optimal value of λ can be found, yielding a set of SNR-designed Maxwell-Boltzmann constellations. Alternatively, the PMF can be adapted to the channel through a numerical search over λ. The latter will be referred to as (channel) optimized Maxwell-Boltzmann constellations.

In the fiber optic channel, the memoryless AWGN assumption does not necessarily hold. The presence of the nonlinear Kerr effect introduces signal dependent distortion through the nonlinear term in Equation (4). The NLI variance from the EGN model models a similar effect, albeit only as a Gaussian noise contribution, where the fourth and sixth order moments of the symbol sequence factors into this variance [6]. An SNR-designed Maxwell-Boltzmann constellation typically results in a constellation shape with larger or equal higher-order moments compared to those of a uniform constellation [3,4]. SNR-designed Maxwell-Boltzmann shaping is thus expected be suboptimal for channels exhibiting strong nonlinear effects [4]. The EGN model can be used to design probabilistic constellation shapes apart from Maxwell-Boltzmann, as in Reference [10], but the assumed noise contributions are still Gaussian.

### 2.4. Kullback-Leibler Divergence as a Measure of Noise Distribution

To quantify the Gaussian noise characteristic and thus explain the effectiveness of different constellation shapes, the Kullback-Leibler divergence (KLD) is used. It is defined as [16]

(6) $$D_{\mathrm{KL}}\left(p,q\right)=\sum_{x}p\left(x\right)\log\left(\frac{p(x)}{q(x)}\right),$$

where *p* and *q* are probability distributions. The divergence is a measure of how one probability distribution differs from another, where a smaller KLD, down to a constant, indicates a closer match between the probability distributions.

KLD will be used to measure the relative distance between the noise distribution, $p_{N}$, and a Gaussian distribution with zero mean and variance matching that of the noise, $N(0,\sigma_{N}^{2})$. This divergence, $D_{\mathrm{KL}}\left(p_{N},N\left(0,\sigma_{N}^{2}\right)\right)$, is interpreted as how much the noise distribution differs from Gaussian or the degree of non-Gaussian noise present in the channel, which will be originating from the nonlinear channel effects. The true noise distribution in a fiber optic channel would in addition depend on the distribution of the input signal, which is not tractable, yet an estimate is needed to evaluate the KLD. For this purpose, an empirical distribution, ${\tilde{P}}_{N}$, is made using the noise samples obtained from received symbols through ${\tilde{P}}_{N}=P\left(Y-X\right)$; this assumes additive and independent noise, but makes no assumption about the noise distribution. The empirical PMF is obtained through a histogram, where complex-valued noise samples are binned into 100 bins for the real and imaginary part each. Through this empirical distribution, we obtain $D_{\mathrm{KL}}\left({\tilde{P}}_{N},N\left(0,{\tilde{\sigma }}_{N}^{2}\right)\right)$, which will be our measure of how Gaussian the noise is.

## 3. Optimization and Evaluation of Probabilistic Constellation Shaping

### 3.1. Iterative Line Search PMF Optimization Algorithm

We shall treat probabilistic shaping of QAM constellations for the nonlinear fiber optic channel through direct optimization of PMFs, using an algorithm conceptually similar to the Blahut-Arimoto algorithm used in References [10,17,18]. The algorithm was first presented in Reference [19] and is summarized in Algorithm 1.

**Figure 1:**
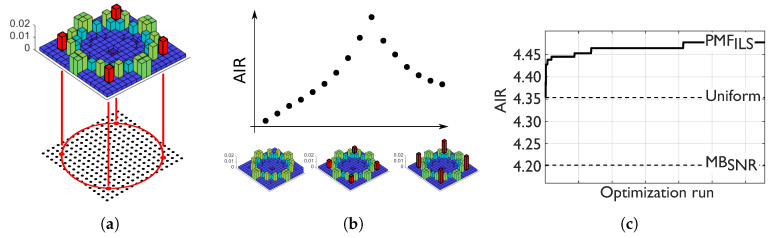
Steps and convergence of Algorithm 1. (**a**) Symbols are selected according to their amplitude, picking out the four red “symbols” marked in the PMF diagram. (**b**) A line seach is performed by altering the probability of occurrence of the selected symbols. (**c**) After iteration over the amplitudes, the algorithm eventually converges on a PMF denoted PMF_ILS_.

To reduce dimensionality and improve robustness over the Blahut-Arimoto algorithm, the algorithm assumes symbols of the same amplitude to have equal probability of occurrence, as seen in the step in Figure 1a. This heuristic will enforce a degree of rotational symmetry given that 90${}^{\circ }$ rotations will be equivalent. The algorithm must be initialized with a PMF and a symbol alphabet. For each set of symbols, with equal amplitude, a line search steps through a range of possible probability of occurrences, in Figure 1b. The AIR for the line search is evaluated using Equation (1) and the auxiliary channel model through Equation (3). The normalization step ensures that the constellation PMF, $P_{A}$, is a valid PMF. The algorithm iterates through the amplitudes a set number of times or until convergence is observed, as seen in Figure 1c. The PMF obtained from Algorithm 1 is denoted as the iterative line search (ILS) PMF, PMF${}_{\mathrm{ILS}}$.

**Algorithm 1:** Greedy mutual information optimization algorithm for constellation PMFs in arbitrary channels.

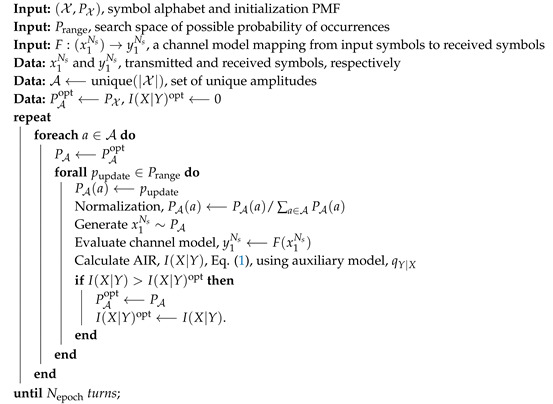



The channel model, *F*, maps from a transmitted sequence, $x_{1}^{N_{s}}$, to a received sequence, $y_{1}^{N_{s}}$, under stochastic influences. The algorithm is agnostic to the exact process of the mapping of *F* and the universal nature of this algorithm stems from this invariance to channel models and modulation formats. For the purposes of this work, a SSFM model and QAM constellation formats are used for all optimizations. In Section 4, where three distinct transmission scenarios are considered, only the definition of the channel model, *F*, changes; definitions are given in the respective sections.

In this work, we also perform numerical optimization of Maxwell-Boltzmann constellations, which is channel agnostic, too, in contrast to SNR-designed Maxwell-Boltzmann constellations, which rely on AWGN channel assumptions. The algorithm is listed in Algorithm 2 and the core principle is the same as the above Algorithm 1; the optimization is instead in a single dimension, rather than multi-dimensional, necessitating only a single parameter (λ) sweep.

**Algorithm 2:** Optimization of Maxwell-Boltzmann constellations.

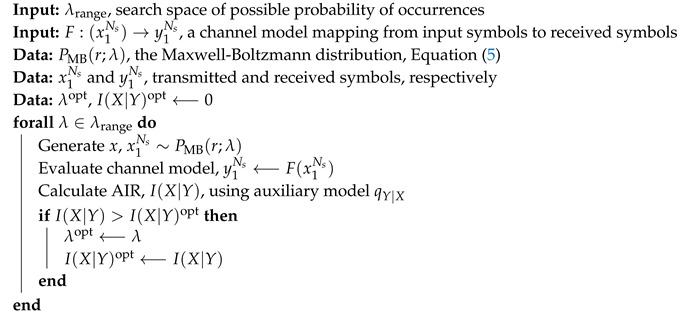



### 3.2. Transmitter and Receiver

**Figure 2:**
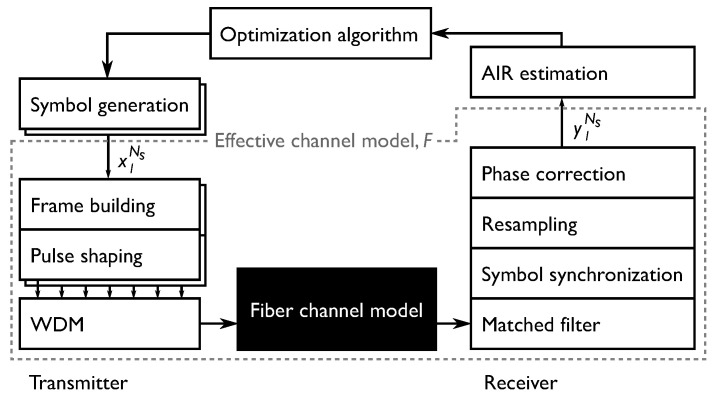
Schematic representation of components of the transmitter, receiver, optimizer and the channel model. Only the channel model changes for the various scenarios and is thus considered a black box.

To evaluate the performance impact of the various constellation PMFs, an optical WDM transmission system is simulated. The test signal is constructed and decoded in the same manner in all cases, but the channel model and signal power into the channel can be changed. A schematic representation of the transmitter, receiver and the relation to the optimizer is depicted in Figure 2. Note that from the perspective of the optimization algorithms, the channel model, *F*, is the whole system after symbol generation and until AIR estimation. All optimizations in the three case studies are performed on 256 QAM constellations in a single polarization. The algorithm is initialized with optimized Maxwell-Boltzmann PMFs according to Algorithm 2.

Going from left to right in Figure 2, the sequence of transmitted symbols are generated directly as realizations of the constellation PMF. The symbol sequence is then pulse shaped using a root-raised cosine pulse with 0.01 roll-off spanning 202 symbols. The single channel baudrate is 32 GBd and 14 such sequences are generated, which are modulated onto 7 carrier frequencies with a spacing of 37.5 GHz and two polarizations. This WDM signal is propagated through the channel under test. Further details on the channel models are given in their respective sections, but all models used in this work are based upon a SSFM implementation with a step size of 1 km. The system, optical fiber and simulation parameters are listed in Table 1.

At the receiver, the signal is filtered using a root-raised cosine filter matching the transmitter’s and then synchronized to the symbol sequence. Frequency domain chromatic dispersion compensation is optionally done before pulse filtering depending on the channel under test. No further equalization is performed on the signal. The mutual information is then estimated for each block using Equation (1). The KLD of the noise, $D_{\mathrm{KL}}\left(P_{N},N\left(0,\sigma_{N}^{2}\right)\right)$, is calculated using the synchronized received symbols and the known transmitted symbols as in Section 2.4.

## 4. Results

Four distinct constellation shapes are evaluated for each channel and across varying power. The constellations are defined by their underlying PMF and are—a uniform constellation; an SNR-designed Maxwell-Boltzmann constellation, which is based upon the AWGN channel assumptions and an effective SNR measurement on a uniform constellation; an optimal Maxwell-Boltzmann constellation selected according to Algorithm 2; and optimized constellation selected according to the iterative line search of Algorithm 1. The constellations will respectively be referred to as: uniform, MB${}_{\mathrm{SNR}}$, MB${}_{\mathrm{Opt}}$ and PMF${}_{\mathrm{ILS}}$.

### 4.1. Dispersion-Managed Systems

**Figure 3:**
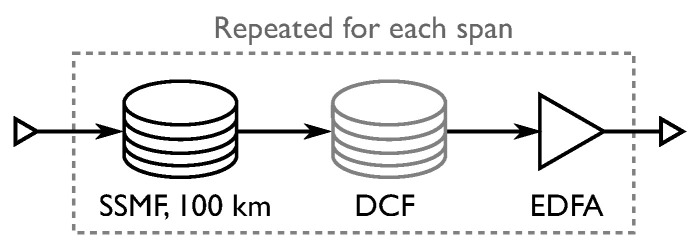
Link setup for the dispersion-managed channel. In the simulations, dispersion management is simulated using ideal filtering.

The simulated dispersion-managed channel consists of four distances of 5, 10, 20 and 40 spans of 100 km fiber, with a link as in Figure 3 replacing the “Fiber channel model” in Figure 2. The inline dispersion compensation is simulated using a linear frequency domain filter, defined by (Reference [20] Ch. 8)

(7) $$H_{f}\left(L,\omega \right)=\exp\left(i\beta_{2}/2\cdot \omega^{2}L\right),$$

where ω is the angular frequency and *L* is the fiber distance propagated. The dispersion compensation is followed by re-amplification to the launch power using EDFA’s with a noise figure of 5 dB. Note that there is no chromatic dispersion compensation at the receiver.

**Figure 4:**
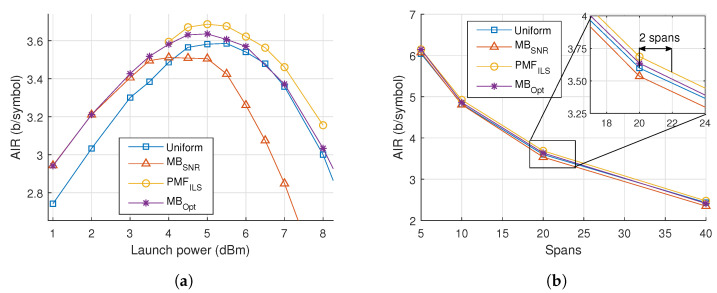
(**a**) Achievable information rate (AIR) versus launch power for 20 spans of a dispersion-managed system. (**b**) Maximum AIR versus number of spans. Detail displays the AIR around 20 spans.

In Figure 4, the results of optimizations and simulations are presented at multiple link distances. After 20 spans, the PMF${}_{\mathrm{ILS}}$ constellations achieve an improvement of 0.1 bits/symbol over uniform and MB${}_{\mathrm{SNR}}$. This distance exhibits a scenario, where MB${}_{\mathrm{SNR}}$ underperforms compared to uniform because of PMF-dependent nonlinear effects. At short distances, MB${}_{\mathrm{Opt}}$ performs close to PMF${}_{\mathrm{ILS}}$, but as the number of spans is increased the performance of MB${}_{\mathrm{Opt}}$ becomes closer to uniform, indicating that the noise distribution is non-Gaussian. The reach gain by PMF${}_{\mathrm{ILS}}$ is 2 span, which is achieved around the 20-span reach.

**Figure 5:**
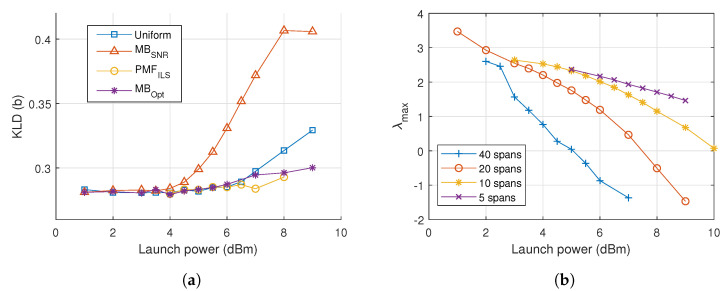
(**a**) Kullback-Leibler divergence (KLD) versus power for 20 spans of dispersion-managed 256 QAM transmission. (**b**) λ of MB_Opt_ versus power for various number of spans.

The noise characteristics are compared across constellations and launch powers by evaluating the KLD of the noise samples relative to a Gaussian distribution, plotted in Figure 5a. Values closer to zero indicate a greater similarity to Gaussian noise. It is seen that that the noise deviates from a Gaussian distribution as the launch power is increased and the choice of connstellation shapes influences this statistic. Especially, the MB${}_{\mathrm{SNR}}$ constellation is associated with a much larger divergence and thus more non-Gaussian noise.

In Figure 5b, the λ of the MB${}_{\mathrm{Opt}}$ constellations are plotted against varying power and number of spans. We note a trend that as power is increased the value of λ decreases, indicating that the PMF becomes less peaked and more uniform. A similar observation can be made with regards to an increasing number of spans. The tendency does not converge towards uniform, but decreases to negative values of λ.

**Figure 6:**
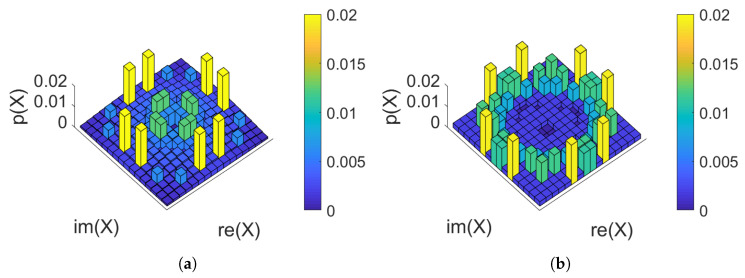
256 quadrature-amplitude modulation (QAM) PMF_ILS_ for 20 span dispersion-managed transmission: (**a**) At the optimum launch power (5 dBm). (**b**) At an elevated launch power (8 dBm). The higher-order moments are summarized in Table 2.

For the 20 span case, two constellations of PMF${}_{\mathrm{ILS}}$ are shown in Figure 6: at the optimum launch power (5 dBm) and at an increased launch power (8 dBm). At 20 spans, the channel in this scenario is significantly non-AWGN and the rate is low enough to make the low-entropy constellations in Figure 6a optimal. The higher-order moments, seen in Table 2, reveal that at the optimum launch power, PMF${}_{\mathrm{ILS}}$ and MB${}_{\mathrm{Opt}}$ both have lower higher-order moments, than MB${}_{\mathrm{SNR}}$. The lowest higher-order moment is obtained with uniform, not the numerically optimized PMFs, demonstrating the need for making a trade-off between linear and nonlinear effects. At elevated launch powers (8 dBm), nonlinear effects dominate and PMF${}_{\mathrm{ILS}}$ results in a ring-like structure and the lowest higher-order moments indicating that the nonlinear effects are stronger.

### 4.2. Unrepeatered Transmission

**Figure 7:**
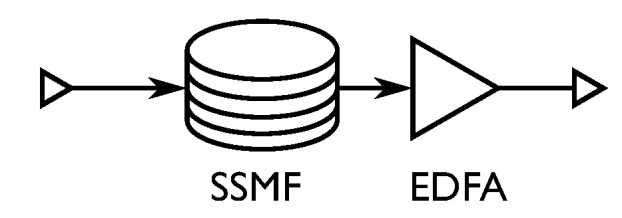
Link setup for the unrepeatered channel.

The unrepeatered links consist of a single span of standard single-mode fiber of 160, 180 and 200 km in length, with a link as in Figure 7. At the end of the span, a single EDFA with a noise figure of 5 dB re-amplifies the signal to the same as its launch power.

**Figure 8:**
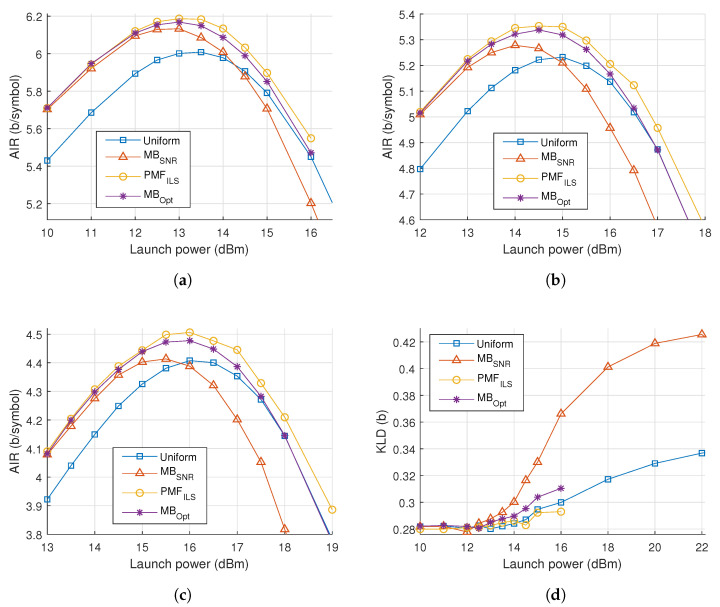
AIR versus launch power in an unrepeatered transmission system with span lengths of (**a**) 160 km, (**b**) 180 km and (**c**) 200 km. (**d**) KLD versus power for a 160 km span length.

In Figure 8a–c, the AIR of the simulated signals can be seen as the launch power is varied over three distances. A maximum shaping gain of 0.2 bits/symbol is obtained at 160 km, with the shaping gain being >0.1 bits/symbol at all distances.

To build upon the results from the dispersion-managed channel, we note the relative performance of the constellation shapes as distance and power is changed. As power and distance is increased, the MB${}_{\mathrm{SNR}}$ constellations degrade in performance compared to the other constellations. Using MB${}_{\mathrm{Opt}}$, an improvement is achieved over uniform, but the improvement diminishes as power is increased and converges to the same performance as uniform. The characteristics of the optimal value of λ mirror those for the dispersion-managed system seen in Figure 5b. Despite being channel tailored, MB${}_{\mathrm{Opt}}$ is intrinsically limited to a specific family of PMFs. PMF${}_{\mathrm{ILS}}$ displays an improvement over both uniform and MB${}_{\mathrm{Opt}}$, even as power is increased beyond the optimum.

**Figure 9:**
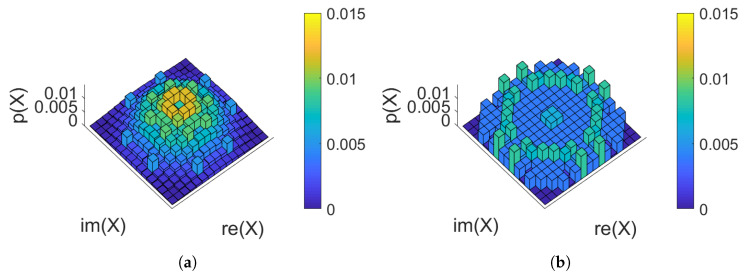
256 QAM PMFILS for 160 km unrepeatered transmission: (**a**) At the optimum launch power (13 dBm). (**b**) At an elevated launch power (16 dBm).

In Figure 8d, the KLD of the noise samples is plotted against power, where the MB${}_{\mathrm{SNR}}$ constellation shaping introduces significant degree of non-Gaussian noise. Both MB${}_{\mathrm{Opt}}$ and PMF${}_{\mathrm{ILS}}$ results in noise characteristics comparable to those produced when uniform PMFs are used for constellation shaping. In Figure 9, the obtained PMF${}_{\mathrm{ILS}}$ are depicted at the optimum and an elevated launch power. We note again a ring-link structure which minimizes the higher-order moments at the elevated launch power.

### 4.3. Channels with Ideal Raman Amplification

**Figure 10:**
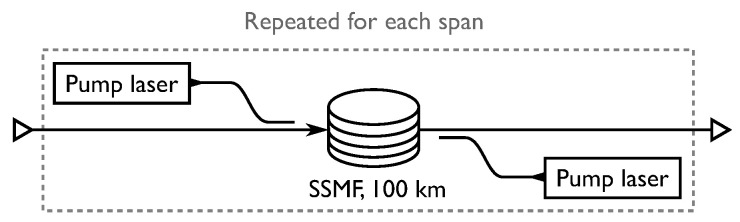
Link setup for the Raman channel. The coupled pump lasers are shown for illustrative purposes only as ideal distributed Raman amplification (IDRA) is simulated.

For raman amplification, a co-propagating laser amplifies the data signal through the process of stimulated Raman scattering, with a setup similar to Figure 10. The Raman model used for the simulations is an IDRA where the signal power is constant with distributed noise contributions as in Reference [8]. Thus the two pumps in Figure 10 are not part of the simulation; instead, the fiber loss, α, is set to zero, thereby removing the loss term from Equation (4).

**Figure 11:**
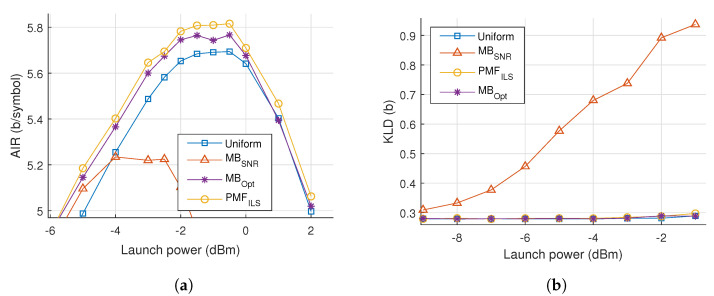
(**a**) AIR versus launch power for 30 spans of fiber with IDRA. (**b**) KLD versus launch power for the same system.

**Figure 12:**
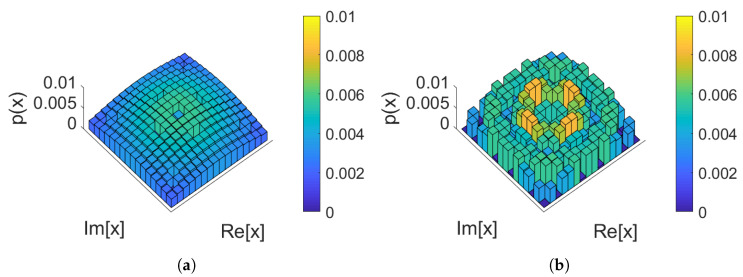
256 QAM PMF_ILS_ for IDRA transmission: (**a**) At the optimum launch power (−0.5 dBm). (**b**) At an elevated launch power (2 dBm).

In Figure 11a, the results of the simulations and optimizations are plotted, where PMF${}_{\mathrm{ILS}}$ yields an improvement of 0.1 bits/symbol compared to uniform. We note that the MB${}_{\mathrm{SNR}}$ constellation severely underperforms on the Raman amplified channel. In Figure 11b, this is backed up by presence of a significant difference in the noise distribution obtained with MB${}_{\mathrm{SNR}}$. This demonstrates the mismatch between the assumptions behind the design of MB${}_{\mathrm{SNR}}$ and the Raman amplified channel, where signal power is effectively constant throughout the entire fiber. In dispersion-managed systems and unrepeatered systems, the signal power attenuates during propagation, gradually decreasing the nonlinear effects. Through MB${}_{\mathrm{Opt}}$ and PMF${}_{\mathrm{ILS}}$ it is possible to recover an improvement over uniform constellations, yet as launch power is increased, MB${}_{\mathrm{Opt}}$ converges to the performance of uniform whereas PMF${}_{\mathrm{ILS}}$ continues to sustain an improvement. The increased degrees of freedom in PMF${}_{\mathrm{ILS}}$ allow it to match the channel characteristics better, compared to MB${}_{\mathrm{Opt}}$ which only has a single parameter. PMF${}_{\mathrm{ILS}}$ is shown in Figure 12 for the optimum power and for an elevated launch power. The ring structure depicted at the elevated launch power minimizes the higher-order moments, indicating the importance of the nonlinear contributions.

## 5. Discussion

In this paper, we investigate the noise characteristics and validity of an AWGN channel assumption in nonlinear optical fiber systems. The particular interest is with regard to constellation shaping and how underlying assumptions about the noisy channel model influence their resulting performance.

At low launch powers, the AWGN model is a close enough approximation of the fiber optic channel that it can be used to design probabilistic constellation shaping solely based upon an SNR measure, since the performances of MB${}_{\mathrm{SNR}}$, MB${}_{\mathrm{Opt}}$ and PMF${}_{\mathrm{ILS}}$ were overlapping, giving evidence that the model assumptions are valid. However, as launch power is increased, it is seen that this model becomes increasingly inaccurate and MB${}_{\mathrm{SNR}}$ degrades to generally having worse performance than the non-shaped uniform constellation.

In the cases presented in this paper, MB${}_{\mathrm{Opt}}$ provided shaping gains through numerical optimization accounting for the channel. This need for channel tailored optimization reflects the effective SNR measure’s dependence upon the PMF in the optical fiber channel, an effect which is also observed in the EGN model. But beyond the optimum power, MB${}_{\mathrm{Opt}}$ converges to a uniform PMF and thus matches the non-shaped performance. For all three nonlinear channel cases in this paper, the noise distribution deviates from Gaussian, as evaluated using KLD, at high launch powers. Thus the optimum launch power lies in a region of increasing nonlinear influence and thus increasing non-Gaussian noise. By expanding the degrees of freedom, PMF${}_{\mathrm{ILS}}$ deviates from MB${}_{\mathrm{Opt}}$ to achieve improved results, leading to a shaping gaing of >0.1 bits/symbol over uniform across all channels. This demonstrates the need for channel tailored constellation shapes in the presented scenarios.

Further improvements could possibly be obtained by allowing the optimization algorithm to operate on higher-order symbols spanning time and polarizations [7].

## 6. Conclusions

Optimization of probablistic constellation shaping is necessary for maximizing the spectral efficiency, and in nonlinear channels, constellation design must account for both nonlinear effects and ASE noise. This is investigated and shown in three cases: dispersion-managed channels, unrepeatered transmission channels and channels with IDRA. Using a channel-agnostic optimization strategy, constellation PMFs can be optimized for the individual, numerically simulated channels. The iterative line search optimization algorithm does not assume the PMFs to belong to a specific family and the increased degrees of freedom allow it to account for non-Gaussian noise effects in the channel.

At low launch powers, the investigated channels behave as conventional AWGN channels, but as launch power is increased, the characteristics of the three channels change. Using the Kullback-Leibler divergence to analyse the noise statistics reveals that SNR-designed Maxwell-Boltzmann constellations increase nonlinear distortion resulting in non-Gaussian noise and thus lead to suboptimal performance in the channels. Despite the differences in length, dispersion-compensation and amplification techniques, the channels share this common characteristic of rendering SNR-designed Maxwell-Boltzmann constellations suboptimal and degrade the performance to worse than uniform as power is increased.

At the optimum launch powers, constellation shapes must balance both nonlinear distortion and ASE noise. This is demonstrated using the iterative line search algorithm, which provides a gain upon numerically optimized Maxwell-Boltzmann constellations. As power is increased further, numerically optimized Maxwell-Boltzmann constellations converge on uniform constellations, because of the increasingly non-Gaussian noise. The channel-tailored constellation shapes achieve gains of >0.1 bits/symbol across all channels and a 2 span reach increase on the dispersion-managed channel, when compared to uniform QAM constellations.

## Figures and Tables

**Table 1:** General system, fiber and simulation parameters.

Parameter	Value
Modulation	256 QAM
Symbol rate	32 GBd
Pulse shape	Root-raised cosine
Roll-off	0.01
WDM channels	7 channels
WDM spacing	37.5 GHz
Polarizations	2
Nonlinear coefficient, γ	1.3 W${}^{-1}$ km${}^{-1}$
Dispersion, *D*	17 ps/nm/km
Attenuation, α	0.2 dB/km
SSFM step size	1 km
Symbols per ch., $N_{s}$	500,000 symbols

**Table 2:** Table of higher-order moments.

	5 dBm		8 dBm	
	$E[|X^{4}|]$	$E[|X^{6}|]$	$E[|X^{4}|]$	$E[|X^{6}|]$
PMF${}_{\mathrm{ILS}}$	1.43	2.44	1.19	1.58
MB${}_{\mathrm{Opt}}$	1.57	3.05	1.25	1.75
Uniform	1.40	2.29	1.40	2.29
MB${}_{\mathrm{SNR}}$	1.91	5.16	1.91	5.14

## Abbreviations

The following abbreviations are used in this manuscript:

AIR	Achievable information rate
ASE	Amplified spontaneous emission
AWGN	Additive white Gaussian noise
EDFA	Erbium-doped fiber amplifier
EGN	Enhanced Gaussian noise
IDRA	Ideal distributed Raman amplification
ILS	Iterative line search
KLD	Kullback-Leibler divergence
MI	Mutual information
NLI	Nonlinear interference noise
PDF	Pobability density function
PMF	Probability mass function
QAM	Quadrature amplitude modulation
SNR	Signal-to-noise ratio
SSFM	Split-step Fourier method
WDM	Wavelength-division multiplexing
